# Presence of the Amphibian Chytrid Fungus *Batrachochytrium dendrobatidis* in Native Amphibians Exported from Madagascar

**DOI:** 10.1371/journal.pone.0089660

**Published:** 2014-03-05

**Authors:** Jonathan E. Kolby

**Affiliations:** One Health Research Group, School of Public Health, Tropical Medicine, and Rehabilitation Sciences, James Cook University, Townsville, Queensland, Australia; Imperial College Faculty of Medicine, United Kingdom

## Abstract

The emerging infectious disease chytridiomycosis is driven by the spread of amphibian chytrid fungus (*Batrachochytrium dendrobatidis*, *Bd*), a highly virulent pathogen threatening global amphibian biodiversity. Although pandemic in distribution, previous intensive field surveys have failed to detect *Bd* in Madagascar, a biodiversity hotspot home to hundreds of endemic amphibian species. Due to the presence of *Bd* in nearby continental Africa and the ecological crisis that can be expected following establishment in Madagascar, enhanced surveillance is imperative. I sampled 565 amphibians commercially exported from Madagascar for the presence of *Bd* upon importation to the USA, both to assist early detection efforts and demonstrate the conservation potential of wildlife trade disease surveillance. *Bd* was detected in three animals via quantitative PCR: a single *Heterixalus alboguttatus*, *Heterixalus betsileo*, and *Scaphiophryne spinosa*. This is the first time *Bd* has been confirmed in amphibians from Madagascar and presents an urgent call to action. Our early identification of pathogen presence prior to widespread infection provides the necessary tools and encouragement to catalyze a swift, targeted response to isolate and eradicate *Bd* from Madagascar. If implemented before establishment occurs, an otherwise likely catastrophic decline in amphibian biodiversity may be prevented.

## Introduction

Amphibian populations are experiencing global decline in response to a storm of assaults including habitat destruction, climate change, and the emerging infectious disease chytridiomycosis caused by amphibian chytrid fungus, *Batrachochytrium dendrobatidis* (*Bd*) [1]–[3]. *Bd* demonstrates low host species specificity and can potentially affect the entire class Amphibia, threatening the survival of thousands of amphibian species [4]. This pathogen can be highly lethal and easily transmissible through direct physical contact with affected individuals or indirectly by exposure to water contaminated with aquatic *Bd* zoospores [5]. Despite infection, certain species can act as reservoir hosts, allowing *Bd* to persist while driving others to extinction. This, together with prolonged environmental persistence provides an optimal situation for pathogen establishment and the collapse of amphibian diversity, especially in aquatic environments [6]–[8].

*Bd* was first identified and described nearly 15 years ago [9], [10], by which time it had already spread to dozens of countries, potentially through the international trade in live amphibians [11]–[13]. Annually, millions of live amphibians are traded globally for the exotic pet trade, biomedical research, and human consumption and this movement of potentially infected animals may be a primary driving force of global *Bd* dispersal [13]–[15]. The transportation of *Bd*-contaminated environmental substrates and field equipment represent additional potential dispersal pathways [6], [16], suggesting that common activities such as freshwater aquaculture and mining may also contribute towards the spread of *Bd* even in the absence of amphibian movement.

Although the spread of *Bd* has continued seemingly unabated for many decades, there remain hotspots of amphibian biodiversity where this devastating pathogen is not yet established and has been presumed absent due to the lack of confirmed field detection, most notably in Madagascar. The first expansive survey for the presence of *Bd* in Madagascar failed to detect this pathogen in 527 amphibians from 79 species sampled from 2005–2006 [17]. To complement this effort, a follow-up survey of 300 animals from 53 species at 12 additional locations were sampled in 2006 and 2007 [18], and a further 56 amphibians from 12 species were sampled in the country's central highlands [19]; all results similarly demonstrated the absence of *Bd* in amphibians sampled despite covering a range of host species and environments, and employing the most sensitive diagnostic tool, the *Bd*-specific quantitative PCR (qPCR) assay. It is remarkable that *Bd* is not already widespread in Madagascar because the country possesses high diversity of amphibians likely to be susceptible to chytridiomycosis, is in close proximity to regions of *Bd* presence in continental Africa (i.e. Tanzania, Malawi, South Africa), and provides high environmental suitability for *Bd* [20], [21].

Thousands of amphibians are exported annually from Madagascar and disseminated globally into the exotic pet trade. An analysis of records obtained from the United States Fish and Wildlife Service (USFWS) through a Freedom of Information Act (FOIA) request shows 39,020 amphibians were exported from Madagascar to the United States between 2006 and 2011, from at least 31 species. Although the international movement of amphibians is believed to help spread *Bd* and thus jeopardize global animal health, we considered access to traded animals a boon to our research goals: demonstration that trade can be approached as an efficient wildlife disease surveillance tool for the rapid detection of *Bd* in Madagascar. The commercial trade in amphibians generates large and diverse sample pools from which proactive surveillance can be performed with less human and financial resources than conventional field surveys. This investigation explored the presence of *Bd* in Madagascar by examining the contents of a shipment of wild-collected endemic amphibians exported directly to the USA.

## Results

In total, 565 amphibians of nine species exported from Madagascar were sampled for *Bd* detection (Table 1). *Bd* was detected in three of 565 animals and each displayed measureable amounts of *Bd* in at least two qPCR replicates, from at least two separate plates. The three species positive for *Bd* were *Scaphiophryne spinosa* (MGSS30), *Heterixalus alboguttatus* (MGHA54), and *Heterixalus betsileo* (MGHB42) (Table 1). Prior to its final qPCR with purified DNA, MGSS30 was tested in two separate qPCR plates and in each, one replicate came up positive; the zoospore loads were 0.332 and 0.040, respectively. When the purified DNA was run a final time, all three replicates of MGSS30 were negative for *Bd*. MGHA54 was tested in two plates prior to DNA purification and again, one replicate per plate came up positive for *Bd*, with zoospore loads of 0.189 and 0.089. After DNA purification, one replicate was again positive; its reported zoospore load was 0.400. MGHB42 was also tested in two plates prior to purification and unlike the other two samples, all six replicates were positive for *Bd*; the average zoospore load for the first plate was 0.395 and the average zoospore load in the second plate was 0.219. After DNA purification, all three MGHB42 replicates were again positive for *Bd*, reporting a mean value of 1.059 zoospores.

**Table 1:** Amphibians from Madagascar sampled for the presence of *Batrachochytrium dendrobatidis* (*Bd*).

Species	No. Sampled	Ulcerations	Sloughing	DOA	*Bd* +	Reference #	ZSE
*Boophis pyrrhus*	58	4	-	11	-		-
*Boophis rappiodes*	39	3	-	1	-		-
*Boophis microtympanum*	65	1	17	18	-		-
*Heterixalus alboguttatus*	78	-	-	1	1	MGHA54	0.089–0.400
*Heterixalus betsileo*	86	1	-	3	1	MGHB42	0.219–1.059
*Dyscophus guineti*	70	1	2	-	-		-
*Scaphiophryne boribory*	31	-	-	5	-		-
*Scaphiophryne madagascariensis*	69	-	-	-	-		-
*Scaphiophryne spinosa*	69	-	-	-	1	MGSS30	0.040–0.332
	565	10	19	39	3		

Conditions potentially indicative of chytridiomycosis were recorded at the time of sampling, including skin ulcerations, sloughing, and death on arrival (DOA). Number of *Bd*-positive samples (*Bd*+) is reported followed by the sample's reference number and range in average zoospore equivalents (ZSE) per run, detected by qPCR.

Examination of amphibians sampled for *Bd* collectively revealed ulcerations (1.8%), heavy skin sloughing (3.4%), and death on arrival (6.9%) in 68/565 animals (Table 1). Because not all deceased amphibians were sampled for *Bd*, the total number of DOA animals was greater with respect to the entire shipment (n = 99; 15.8%). No such conditions were observed in any of the three *Bd*-positive amphibians at the time of sampling.

## Discussion

The presence of *Bd* has been confirmed in Malagasy amphibians for the first time. These amphibians were collected from the wild for the pet trade, exported to the USA and sampled immediately upon arrival. One sample produced a strong signal for *Bd* presence (MGHB42), and two others displayed weak indications: MGSS30 and MGHA54. Despite the low intensities, these two samples certainly displayed positive signals and, most important from separate plates, suggesting the signals were real and not due to contamination from the positive controls. It is not uncommon for the standard controls to contaminate a single replicate, but to do so across multiple plates, has never been witnessed and is unlikely. After DNA purification, all three MGSS30 replicates were negative for *Bd*. This is perplexing and could suggest that the original DNA aliquot used in the first two plates (prior to purification) was contaminated. However, it is also possible that the particular aliquot of DNA used in the final run did not actually contain *Bd* DNA, although it existed in the sample; MGSS30's measured zoospore loads were incredibly low and give some credence to this possibility. MGHA54, like MGSS30, similarly never had all replicates within a single plate turn up positive. Its zoospore load was similarly low, again suggesting that *Bd* DNA similarly might not have been present in each replicate. Because a single replicate was positive from three different plates, including the final qPCR using purified DNA, these data do suggest MGHA54 was positive for *Bd*. Although it is difficult to discern the truth about MGSS30 and MGHA54, because separate plates yielded positive replicates and contamination was unlikely, I report these samples as *Bd*-positive. Regardless, all nine replicates of MGHB42 were positive for *Bd*, undeniably confirming its presence in material from Madagascar.

The status of *Bd* in wild amphibian populations in Madagascar remains uncertain and calls for urgent targeted field surveys in regions where these *Bd*-positive animals were likely collected. The human-assisted movement of traded animals introduces an opportunity for *Bd* cross-contamination between species and collection origins prior to exportation if animals are housed in shared enclosures where direct or indirect contact is allowed. Accordingly, transmission of *Bd* between Malagasy species from different collection localities may potentially exaggerate the number of affected species in wild populations and suggested distributional range of infection.. Furthermore, identifying the source of *Bd* detected in traded animals becomes especially challenging when animals from multiple countries are also present in the trade sector. Fortunately, this is not the case in Madagascar; commercial amphibian importation does not occur in the country [17] and only those of national origin are traded. The absence of foreign-sourced amphibian species suggests my detection of *Bd* is not simply an artifact of re-exportation through the amphibian trade, but instead a reflection of *Bd* presence in the wild in Madagascar. Still, other non-amphibian wildlife trade activities may unknowingly introduce foreign infectious material to Madagascar and expose wild-collected frogs prior to exportation if housed at a shared facility (i.e. exposure to *Bd*-contaminated water accompanying freshwater fish importations). Albeit unlikely the result of such cross contamination, I employed a conservative approach by interpreting these data as confirmation of *Bd* presence in Madagascar within the amphibian trade, but not yet irrefutable evidence for *Bd* presence in wild amphibian populations, despite the strong suggestion.

A second, more specific tier of surveillance via targeted field sampling applying this new information, is now imperative to determine the current extent of *Bd* in Madagascar outside the trade sector. A predictive model of *Bd* distribution [20] shows that the highest climatic suitability for *Bd* overlaps particularly closely with the distributional range of *H. betsileo*, from which MGHB42 was collected. Interestingly, the distributions of *H. albuguttatus* and *S. spinosa* fall on the periphery of this climatic range and may have been collected from areas with moderate to low *Bd* suitability, potentially explaining their exceptionally low *Bd* zoospore loads compared to that detected in the specimen of *H. betsileo*. Accordingly, surveys to trace back the source of the *Bd* detected herein should commence immediately within the distributional ranges of *H. betsileo*, *H. alboguttatus*, and *S. spinosa*, target the larvae and subadults expected to exhibit increased susceptibility to infection, and include bioregions suitable for *Bd* [22] to maximize the chances of rapidly detecting *Bd* if currently present in wild populations.

The lack of *Bd* detection in previous field surveys of wild amphibians [17]–[19] and these newly reported *Bd*-positive animals suggest the presence of *Bd* in Madagascar is a recent phenomenon and not yet widespread. *Bd*-related die-offs have not been documented and infection prevalence is expected to still be extremely low in wild populations, if currently affected. The conditions necessary to result in *Bd* establishment in amphibian populations following exposure are poorly understood. Infection with as little as one *Bd* zoospore can result in chytridiomycosis [5], and affected amphibians have been observed to release 68 infectious *Bd* particles per minute when in an aquatic environment [23]. Therefore, the detection of *Bd* in *H. betsileo* and *H. alboguttatus* is especially concerning because these species breed in both permanent and temporary water bodies and an outbreak in wild populations may both promote extended environmental persistence and facilitate indirect transmission to nearby aquatic species, increasing the opportunity for pathogen establishment. The spread of *Bd* can occur rapidly following introduction to a naive region, estimated as much as 25–282 km/y [24], and the data presented herein provides impetus to quickly reevaluate the presence of *Bd* in Madagascar.

The confirmation of *Bd* in amphibians exported from Madagascar presents an opportunity to intervene prior to the first confirmed outbreak in wild populations – an outbreak with potentially irreparable ecological consequences. It is no longer questionable whether or not *Bd* will become introduced to Madagascar; it is now a tangible threat. Survival of the country's amphibians now requires an efficient network of proactive surveillance and rapid response to quickly identify additional introduction events and minimize exposure to wild populations [25], because pathogen eradication is considered implausible following establishment. The provenance of *Bd* detected in this investigation remains an enigma, especially considering the absence of commercial amphibian trade into Madagascar, suggesting a more insidious mechanism is responsible for the introduction. Accordingly, *Bd* may continue to arrive in Madagascar and creep closer towards establishment until the true introduction pathway is identified, targeted and controlled. Early detection now provides the opportunity to interrupt pathogen establishment, but if not acted upon with haste, disease-associated ecological decline in Madagascar may soon become inescapable.

## Materials and Methods

### Ethics

Amphibians were imported under United States Fish and Wildlife Service (USFWS) License No. LE65317A-0 and accompanied by a cleared USFWS Declaration for Importation of Wildlife (Form 3-177). None of the species included in this investigation are currently protected or endangered and therefore, no additional special permits were necessary. Permission to export the amphibians was granted by the Government of Madagascar with permit #'s 017/12-MEF/SG/DREF.ATS/EXPORT and 018/11-MEF/SG/DREF.ATS.

### Amphibian Sampling

In February 2012, a shipment containing 565 wild-collected amphibians from Madagascar was exported and sampled for the presence of *Bd* upon importation to the United States. Of 17 endemic amphibian species commercially available from this particular supplier, nine were systematically selected for sampling to represent a potentially wide coverage of biogeographical regions and altitudinal ranges where *Bd* was expected to thrive if present. These decisions were made by comparing species distribution maps provided by the IUCN Red List of Threatened Species [26] with work that identified a predicted region of optimal *Bd* survival based on climatic suitability [20]. Sampling priority was accordingly directed towards species with distributions that overlap this high risk zone, in addition to those vulnerable to *Bd* exposure based on life history characteristics, most importantly association with aquatic habitats [22].

Following regulatory clearance for importation into the USA, the shipment was collected from the airport and immediately transported to a small greenhouse specifically constructed to provide a controlled area for receipt and sampling of these animals. This structure had no previous exposure to amphibians and all interior surfaces were first washed with a 10% bleach solution to minimize any potential risk of domestic *Bd* contamination. The shipment remained sealed for the duration of transport from Madagascar to the USA, and was not opened until first secured inside this sampling location to further prevent opportunities for contamination. All contents of the shipment were handled exclusively with fresh pairs of Nitrile gloves.

**Figure 1:**
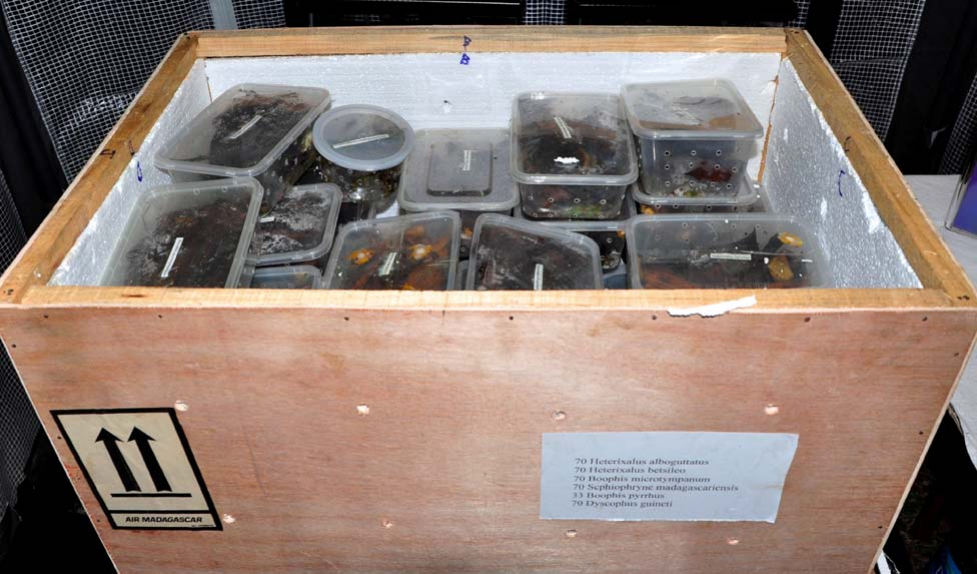
One of two crates of amphibians sampled for *Batrachochytrium dendrobatidis* upon arrival from Madagascar. Amphibians were shipped sealed in wooden crates, insulated with 1/4″ Styrofoam, and packed in plastic containers filled with damp sphagnum moss and leaves.

Amphibians arrived inside two wooden crates insulated with 1/4″ Styrofoam (Fig. 1). Within these crates, the amphibians were packed in plastic containers filled with damp sphagnum moss and leaves as bedding material. Some containers housed multiple amphibians whereas others were packed individually; this varied by species and size of amphibian, but containers housing multiple individuals did not combine species. All amphibians were adults, with the exception of *S. spinosa*, for which only subadult frogs were received. Upon opening the crates, containers were arranged by species and the contents of each examined and sampled for the presence of *Bd*. A sterile fine-tipped rayon swab (Medical Wire & Equipment Co., MW113) was drawn across each amphibian's hands, feet, and pelvic patch five times each. Samples were stored in 2 mL vials filled with 1 mL 70% ethanol as preservative. To prevent cross-contamination between samples, fresh pairs of Nitrile gloves were changed each time a new amphibian was handled.

Each animal was examined immediately prior to swabbing and its condition recorded. Potential clinical symptoms of chytridiomycosis were noted, including the presence of skin ulcerations, skin sloughing, and death [27]. Most specimens of the nine target species were sampled, except for dead animals that arrived in advanced stages of decomposition (n = 60), which were excluded from this investigation. All live amphibians were swabbed individually. When multiple dead animals arrived in the same container, a single swab was used to sample all carcasses; this maneuver increases cost-efficiency of analysis, resulting in fewer swabs (n = 551) than the total number of animals actually sampled (n = 565). Following the prompt completion of sample collection for this investigation, all live amphibians were transferred back into the course of the domestic pet trade.

### Molecular Analysis

Thirty swabs deemed as high priority, those most suspect of *Bd* infection based on physical examination, were first immediately shipped to the San Diego Zoo Amphibian Disease Laboratory for testing. Samples were processed via a sensitive quantitative PCR assay (qPCR) specific to *Bd* following standard methods [28], [29]. Assays were run on an Applied Biosystems 7900HT thermocycler using 384 well plates with Applied Biosystems exogenous internal positive control labeled with Vic in separate wells to test for the presence of PCR inhibitors. For each sample, 5 µl of 1∶10 dilution of swab DNA was added to each well for a final total qPCR volume of 20 µl. Standard curves were generated with 10-fold serial dilutions (range 10,000 to 0.001 zoospores) of laboratory cultivated *Bd* zoospores.

The remaining 521 swabs were also processed via qPCR according to established protocols [29]–[31] at Yale University. Samples were extracted with 150 µl Prepman Ultra (Applied Biosystems, California, USA), with a final 30 µl of supernatant removed for downstream use. An aliquot of this supernatant was diluted 1∶10 in DNase-free water for qPCR. The qPCR protocol used SensiMix II Low Rox (Bioline, Massachusetts, USA) as the qPCR master mix [32]. Samples and controls were run in triplicate with three positive, standard control samples (100, 10, and 1 zoospore/well, made from JAM81 pure culture; see Boyle et al. 2004 for standard control construction) and one non-template control (DNase free, molecular-grade water). When the qPCR assay failed to detect *Bd* in all replicate wells, the sample was deemed negative for *Bd*. When one of three replicates successfully detected *Bd*, the sample was rerun (in triplicate again) in a subsequent plate. For rerun samples that had at least a total of two of six replicates positive for *Bd* (from at least two separate plates) or samples that had *Bd* in all replicates, the original DNA supernatant stock was purified and tested, at full-strength, in a final qPCR. Full-strength DNA from PrepMan Ultra inhibits qPCR, so this DNA must be cleaned-up or diluted prior to use [30]. The remaining full-strength DNA was purified using Performa DTR Gel Filtration Cartridges (Edge Biosystems, Maryland, USA). The cartridges were loaded and prepared by spinning at 750× g for two minutes. The remaining DNA was added to each column, loaded directly onto the gel matrix, and then spun for two minutes at 750× g. Five microliters of this eluted, purified, full-strength DNA was loaded into three replicate wells in a final qPCR (i.e., this DNA was not further diluted prior to qPCR). All zoospore loads described in this report have not been converted; here, reported zoospore loads come from 5 µl DNA (1∶10 or full-strength), placed into 20 µl reaction volumes.
